# Social network- and community-level influences on contraceptive use: evidence from rural Poland

**DOI:** 10.1098/rspb.2015.0398

**Published:** 2015-05-22

**Authors:** Heidi Colleran, Ruth Mace

**Affiliations:** 1 Institute for Advanced Study in Toulouse, Toulouse School of Economics, 21 allee de Brienne, Toulouse 31015, France; 2 Department of Anthropology, University College London, 14 Taviton Street, London WC1H 0BW, UK

**Keywords:** contraception, social transmission, social networks, community effects, fertility decline, cultural evolution

## Abstract

The diffusion of ‘modern’ contraceptives—as a proxy for the spread of low-fertility norms—has long interested researchers wishing to understand global fertility decline. A fundamental question is how local cultural norms and other people's behaviour influence the probability of contraceptive use, independent of women's socioeconomic and life-history characteristics. However, few studies have combined data at individual, social network and community levels to simultaneously capture multiple levels of influence. Fewer still have tested if the same predictors matter for different contraceptive types. Here, we use new data from 22 high-fertility communities in Poland to compare predictors of the use of (i) any contraceptives—a proxy for the decision to control fertility—with those of (ii) ‘artificial’ contraceptives—a subset of more culturally taboo methods. We find that the contraceptive behaviour of friends and family is more influential than are women's own characteristics and that community level characteristics additionally influence contraceptive use. Highly educated neighbours accelerate women's contraceptive use overall, but not their artificial method use. Highly religious neighbours slow women's artificial method use, but not their contraceptive use overall. Our results highlight different dimensions of sociocultural influence on contraceptive diffusion and suggest that these may be more influential than are individual characteristics. A comparative multilevel framework is needed to understand these dynamics.

## 1. Introduction

The global fertility decline that is now unfolding is one of the most profound social trends of the last 200 years. However, classic evolutionary and economic models have struggled to predict variation in the onset or persistence of low fertility [1–4], generating increasing interest in ideational diffusion as a major driver of fertility decline. The diffusion of ‘modern’ contraceptives—as a proxy for the spread of low-fertility norms—is of interest to researchers across the social sciences [5–11]. Access to modern contraceptives is thought to reveal what demographers call an ‘unmet need’ for effective fertility limitation, driving significant fertility declines and justifying huge investments in family planning programmes around the world [12,13]. But it remains unclear how contraceptive behaviour spreads in a population [7,8,13–15], or whether different factors account for the diffusion of alternative method types.

Modern contraceptive uptake tends to be socially and culturally clustered, ignoring political and economic boundaries, spreading readily between co-religionists and groups sharing a common linguistic or ethnic affiliation [1,14–16]. Inferring individual decision-making mechanisms from these macro-level patterns remains a significant challenge, with greater integration needed between the individual characteristics that proxy women's economic and social learning opportunities and the broader sociocultural dynamics that additionally constrain or facilitate contraceptive diffusion. Wealthy and educated women routinely adopt modern contraceptive practices with a higher probability [17,18], as do those exposed to outside ideas via migration [19], acculturation [20], the mass media [21,22], and social and kinship ties [19,23]. But individuals also frequently adopt modern contraception at lower wealth or educational levels than would be expected if these were the only factors that matter [6,15,24–26]. Social interactions within networks [1,2] and communities [6,15,26–28] are important additional influences on contraceptive uptake. Understanding the nested structure of contraceptive decision-making can help determine the extent to which observed behaviour reflects purposive information seeking, or a form of social or ideational contagion. This understanding is important because socially structured interactions may scale up to have important demographic impacts, generating unexpected patterns in contraceptive prevalence [27,29,30] or lower levels of uptake than would be predicted if access to information is all that matters.

Cultural evolutionary theory provides a framework for understanding behaviour change as an interaction between evolved individual decision-making heuristics (e.g. ‘copy when uncertain’) and population-level processes (e.g. cultural selection, drift), existing trait distributions, or cultural sub-structures that may affect the rates and modes (channels) of social transmission. This approach integrates individual characteristics and learning strategies with the dynamics of shifting social norms. Theoretical work predicts, for example, that a conformist bias promotes homogeneous behaviour within groups [31] driving both the initially slow and later rapid uptakes typical of innovation diffusion [31,32] (though see [33]). When information about the pay-offs to a behaviour is uncertain or costly to acquire, prestigious or successful individuals may disproportionately influence decision-makers [34]. The ‘background’ characteristics of a population, such as mean education, can accelerate rates of horizontal and oblique social transmission, de-emphasizing vertical and conformist transmission [30,35–37]. Combined with population sub-structure [30,38,39], which constrains whom individuals observe and interact with, the multilevel dynamics of social transmission can allow culturally or genetically ‘suboptimal’ traits, such as ineffective medicines [40] or low-fertility norms [30,41], to diffuse and be maintained in a population. An important insight is that a balance of information ‘producers' and ‘scroungers' (i.e. asocial and social learners) is necessary for populations to adaptively track changing environmental circumstances [38,39]. This generates the expectation that socially transmitted information and ‘copying’ behaviour will be more important than will purposeful information seeking when environmental change is moderately rapid, but not too rapid, and less important when change is extremely fast or slow [38] (see also [39]).

This approach can cohere disparate sources of empirical evidence on contraceptive uptake in the context of demographic transitions. For example, opinion leaders [42–45] and central social network partners have been shown to disproportionately influence other women's contraceptive use and ideation [43–46]. The particular contraceptive methods that communities end up endorsing are often highly path-dependent [27,29], and when women rely on their social networks for contraceptive information, they may not adopt the most effective methods [29]. Where economic development is slow, or high fertility desired, modern contraception does not seem to spread at all, despite a long exposure and access to reliable information through health clinics and other formal outlets [7,8,28]. Diffusion of information is therefore not equivalent to diffusion of behaviour. Relatively little is known empirically about the likely mechanisms by which individuals socially learn [47–49], for example, whether frequency-dependent dynamics in ego-networks or the contraceptive status of particular family members or friends drives the odds of using contraception [7,8,50]. Threshold effects have been shown to be important [50,51], such that beyond a small number of social models using contraception, additional users in the network had marginal or insignificant effects on contraceptive uptake [50]. The size, composition and density of ego-networks have also been shown to play a role in contraceptive use [51,52] by enabling rapid dissemination of new information that facilitates behaviour change or by strongly reinforcing anti-contraceptive norms [2,31].

But network influences may be hard to detect for the early adopters of contraceptive innovations [7,8]. Individual or network characteristics alone therefore provide only a partial picture of the social structure of reproductive decisions. Integrating these with community-level characteristics may help capture wider social influences and allow a wider range of cultural evolutionary hypotheses to be tested. These include how features of the wider social group, such as the average education or religious commitment of inhabitants, may alter the diffusion of novel cultural traits. Community-level studies have shown that the local prevalence of contraceptives and the behaviour of neighbouring individuals can accelerate contraceptive uptake [15,24] and fertility decline [25,36], potentially by altering social network interactions or the rates of different modes of social transmission (e.g. by accelerating horizontal relative to vertical transmission in a population [30,35]). However, most available sources of demographic data are nationally (rather than locally) representative, aggregating across women's private social networks and local villages or towns. Few studies have been able to examine the appropriate predictors at individual, kin-group, social network and community levels that are needed to simultaneously capture multiple levels of influence.

A further complication is that research on contraceptive uptake is typically carried out in developing populations where educational and economic opportunities for women are often limited, and where modern contraceptives may be used to space reproductive events rather than to reduce family sizes [7,53,54]. So modern contraceptives do not always clearly proxy the adoption of low-fertility norms. It is important to establish whether modern contraceptives are uniquely predictive of fertility decline by comparing how different methods are used and spread in the same context, because the diffusion dynamics may differ between method types. If alternative methods are similarly effective and less locally taboo, they may face fewer barriers to cultural transmission and disseminate more easily.

Here, we examine contraceptive use in a high-fertility population in rural Poland, where a wide variety of contraceptive methods are available, where women have a wide range of economic and educational opportunities, and where fertility is rapidly declining [36]. Using new data, we compare the multilevel predictors of two different measures of contraceptive use: (i) any method and (ii) ‘artificial’ methods only. This distinguishes a general proxy for a desire to control fertility from a subset of more culturally taboo methods, which may be subject to different cultural transmission biases. Our comparative approach allows us to ask the following fundamental questions: (a) Are artificial methods better proxies for the decision to reduce fertility? (b) Are sociocultural factors more important for some than for other methods? (c) Are community-level effects different for different method types? Using multilevel regression to account for the structure in our data, and likelihood-based model selection [55] to quantify the strength of evidence in favour of competing models, we show that: (a) fertility decline to date in this population has primarily been achieved via natural family planning methods (NFP); (b) sociocultural predictors, especially the contraceptive status of female kin and friends, have the strongest and most consistent effects on contraceptive use; and (c) community-level characteristics are independently associated with contraceptive use. Women living in highly educated communities have higher odds of using contraceptives in general, but not artificial methods, whereas women living in highly religious communities have lower odds of artificial method use, but are not less likely to have used contraceptives in general.

Our data come from interviews with 1995 randomly selected women (aged 18–91) living in 22 randomly selected communities in the midst of a demographic and subsistence transition in southern Poland (electronic supplementary material). The data capture a period of profound social and economic change in one of the few European regions where subsistence farming is still practised and where fertility and contraceptive use are highly varied. The study region has a long history of peasant agriculture, with more than 65% (1255) of respondents living in households subsisting partly or mainly from their own produce. Following Poland's rapid transition to a market economy in the early 1990s and accession to the EU in 2004, small-scale farming is being abandoned in favour of wage-labour [56], and fertility is declining [36]. Nonetheless, completed fertility in our sample is dramatically higher than Polish national estimates, with a mean of 3.8 (s.d. 2.15) children per woman, which varies significantly across communities [36].

**Figure 1.**
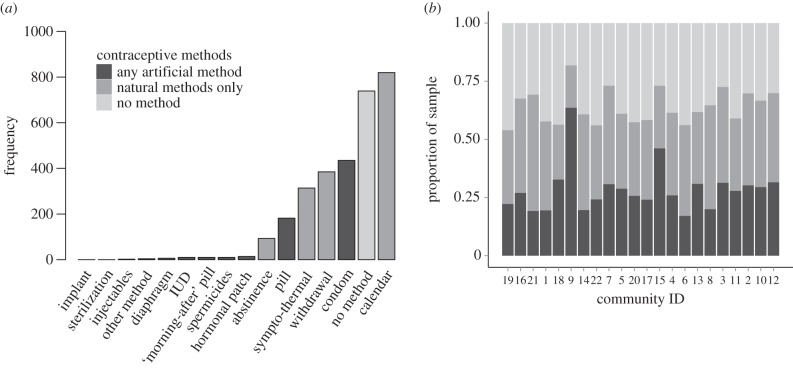
Frequency distributions of (*a*) contraceptives ever used by women in the sample (*n* = 1972) and (*b*) proportion of women using the different method types. Bars in (*b*) are ordered in terms of increasing population density from left to right. Community IDs give the order in which the communities were sampled.

These communities are ethnically and religiously homogeneous, with near-universal observance rates (98% practising Catholics, compared with a national average of 86.9% [57]). Nonetheless, methods for controlling fertility are varied (figure 1) and have been used in all age cohorts (electronic supplementary material, table S3). We collected data on the use of 15 different contraceptive methods (figure 1) by respondents, their female kin and up to five female social network partners or ‘alters’ (electronic supplementary material, table S1). We distinguish between ‘natural’ and ‘artificial’ contraceptives, as opposed to the ‘modern’ versus ‘traditional’ dichotomy. Sixty-three per cent of our sample (1248 women) used some form of contraception in their lifetime and figure 1*a* shows that natural ‘fertility awareness' methods are more highly represented than are artificial methods, with the calendar (rhythm) method being used by 41% (820) of all women. Twenty-eight per cent (544) of women have used an artificial method, with the remaining 37% (724) having never used any method at all (note that women may have used multiple methods). Figure 1*b* shows the proportion of women who have ever used an artificial method (black), who use natural methods only (dark grey) and who use no methods at all (light grey) in each of the 22 communities, in order of increasing population density. This immediately shows that artificial methods are not more highly represented in more densely populated communities.

The Catholic Church discourages artificial contraceptives, but NFP is discussed during compulsory pre-marital courses. Respondents young and old expressed strong norms about appropriate contraceptive use, with fertility awareness typically considered appropriate at any age and artificial methods inappropriate prior to at least the first child's birth. As in many countries, conversation about contraception is loaded with controversial social and political themes, and extra-marital discussion is limited to women's closest friends and kin, giving them significant influence over decision-making. Community leaders build and maintain local consensus about reproduction, additionally constraining conversation about fertility control. Given the prevalence of contraceptive use in our sample, it appears that women are able to gain information on and experiment with methods that may be difficult to discuss publicly. Our study aims to examine which predictors most strongly and consistently affect this behaviour, and whether different factors matter for different contraceptive types.

Our multilevel logistic regressions use the same predictors and the same sample of women (23 women living outside the study communities were excluded, leaving a sample of *n* = 1972) in the 22 communities, with the dependent variable entered as a binary outcome (0 = never used, 1 = ever used). We group predictors into predefined classes, broadly representing different kinds of incentives and influences. We incorporate only well-supported covariates in the existing literature, and do not remove non-significant variables. Starting with (1) life-history controls (LH model): *age*, *age*^2^, *age*^3^, *parity* (number of children), *marital status* and *experience of under-five child mortality*, we add (2) individual socioeconomic predictors (SES model): *farmer status*, *education*, *household material wealth*, *household farming wealth* and *household market integration*; (3) individual sociocultural characteristics (IS model), i.e. predictors characterizing the propensity to use contraceptives based on individual exposure to ‘outside’ ideas or to religiosity: *migrant status*, *exposure to social media* and *religiosity*; (4) social network and kin effects (SN model): *contraceptive status* (ever-user of the relevant method) *of mothers*, *sisters*, *other female kin* and *frequency of ever-users among female ego-network partners*, including controls for *network size* and *proportion of kin in the network* and (5) community-level predictors (CL model): *mean education* in the community and *mean religiosity*, controlling for presence of a *health centre* and a *church*.

Our multilevel models compare the same predictors on different outcome categorizations, and our model comparisons assess the weight of evidence in favour of different classes of predictors. This approach follows recent research on fertility decline by explicitly comparing different model classes [58], but differs in not performing a first-stage variable selection. This is because our interest is in a comparative understanding of the multilevel predictors of different outcome parametrizations rather than in finding the most parsimonious set of variables to explain each outcome.

## 2. Results

### (a) Comparison of reproductive outcomes for users and non-users of contraception

Post-reproductive women who had used artificial contraceptives did not have significantly fewer children than non-users of these methods (table 1). Rather, the important differences are between users of any method versus never-users. Post-reproductive users of any method had significantly fewer children, as well as significantly earlier ages at last birth, shorter reproductive spans and lower rates of under-five child mortality. There are no significant differences in age at first birth, age at marriage or the length of the first inter-birth interval. This suggests that contraceptives have been primarily used for stopping rather than for early spacing of reproduction. Further breakdowns reveal that fertility differences are mainly driven by sympto-thermal and calendar methods (electronic supplementary material, table S4). Fertility declines to date have therefore been achieved through NFP rather than through artificial methods.

**Table 1.** Comparative reproductive parameters of users and non-users of (i) any method of contraception and (ii) artificial methods. CFS, completed family size; AFM, age at first marriage; AFB, age at first birth; ALB, age at last birth; fIBI, length of first inter-birth interval (months); RS, reproductive span (years); u5M, experience of under-five child mortality (yes or no). All comparisons are either Welch *t*-tests assuming unequal variances (continuous variables) or Fisher's exact tests (dichotomous variables).

	CFS (±s.d.)	*n*	AFM (±s.d.)	*n*	AFB (±s.d.)	*n*	ALB (±s.d.)	*n*	fIBI (±s.d.)	*n*	RS (±s.d.)	*n*	u5M (±s.d.)	*n*
(i) any method														
non-users (45+)	4.00 (2.37)	455	22.76 (4.47)	426	23.74 (4.08)	422	33.12 (10.01)	455	26.78 (25.28)	455	9.62 (6.13)	455	0.08 (0.27)	455
users (45+)	3.62 (1.88)**	452	22.73 (3.42)	443	23.79 (3.78)	439	30.14 (7.73)***	452	27.77 (22.41)	452	8.47 (5.87)**	452	0.03 (0.16)***	452
(ii) artificial methods														
non-users (45+)	3.84 (2.18)	833	22.79 (4.04)	798	23.83 (3.97)	790	31.91 (8.97)	833	27.25 (23.51)	833	9.13 (6.45)	833	0.06 (0.23)	833
users (45+)	3.51 (1.76)	74	22.25 (3.40)	71	23.03 (3.35)	71	30.18 (8.44)	74	27.52 (27.96)	74	8.08(5.70)	74	0.03 (0.16)	74

*p < 0.05, **p < 0.01, ***p < 0.001.

### (b) Comparison of parameter estimates on the different measures of contraceptive use

**Figure 2.**
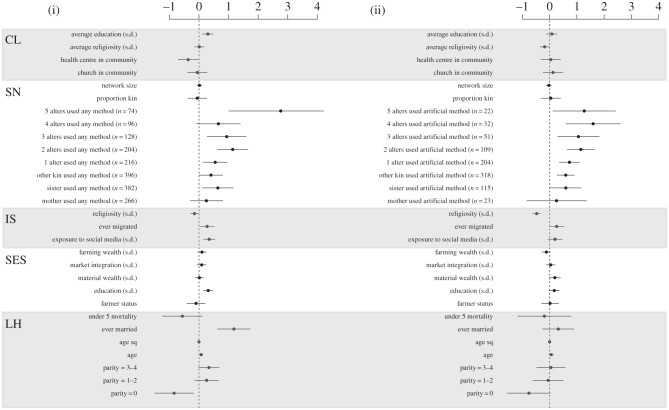
Multilevel logistic regression estimates of the use of (i) any and (ii) artificial contraceptives. Plots show *β* coefficients (points) and 95% CIs (bars) from the global models. Predictors are grouped into theoretically defined classes; LH, life-history parameters; SES, socioeconomic predictors; IS, individual sociocultural predictors; SN, social network and kin effects; CL, community-level effects. Reference categories: parity 5+; 0 alters used contraceptives.

Individual life-history and socioeconomic characteristics are inconsistent predictors of contraceptive use (figure 2, LH and SES models). Married women and those with intermediate numbers of children have elevated odds of using any method, but not of artificial methods. Experience of under-five child mortality is negatively but not significantly associated with any contraceptive use and is not associated with artificial contraceptive use. Childless women are less likely to have used contraception in general, but the effect is less pronounced for artificial than for any methods. Educated women are more likely to use contraceptives in general, but, surprisingly, do not have strongly elevated odds of using artificial methods. Materially wealthy women are slightly more likely to use artificial methods, but they are not more likely to use contraceptives in general, potentially indicating economic barriers to artificial contraceptive use. None of the other individual-level socioeconomic characteristics are important predictors of either kind of contraceptive use.

Individual sociocultural predictors are somewhat more consistent in their associations with contraceptive use. Migrants and women with higher levels of exposure to social media have significantly higher odds of using any contraceptive types, but not artificial methods (figure 2, IS model). Religious women are consistently less likely to use contraceptives overall, and the effect size is more than three times larger for artificial methods (37% lower odds; OR = 0.63, 95% CI = 0.55, 0.73) per 1 s.d. increase in religiosity than for any methods (12% lower odds; OR = 0.88, 95% CI = 0.77, 1.00) per 1 s.d. increase.

Social network and kin effects are strikingly similar for both types of contraceptive use and have dramatically larger effect sizes than all other variables in both models (figure 2, SN model). Women with contraceptive-using sisters and other female kin, but not including mothers, have substantially elevated odds of having used all types of contraceptives. Odds for using any method are 88% higher when a sister used (OR = 1.88, 95% CI = 1.12, 3.15) and 48% higher when other female kin used (OR = 1.48, 95% CI = 1.00, 2.18). Odds for artificial method use are 81% higher when a sister used (OR = 1.81, 95% CI = 1.03, 3.17) and 78% higher when other female kin used (OR = 1.78, 95% CI = 1.30, 2.43). The frequency of contraceptive users in a woman's ego-network is also strongly associated with increased odds of using both types of method in a broadly linear trend, with the magnitude of the effect generally increasing with additional contraceptive users. Compared with women with no users in their networks, the odds of using any method are 72% higher when there is one network user (OR = 1.72, 95% CI = 1.16, 2.56); more than tripled for two users (OR = 3.11, 95% CI = 1.88, 5.14); approximately 2.5 times higher for three users (OR = 2.49, 95% CI = 1.30, 4.76); 84% higher for four users (OR = 1.84, 95% CI = 0.89, 3.81); and increased by a factor of more than 15 for five users (OR = 15.14, 95% CI = 2.70, 84.82). The odds of being an artificial method user are doubled when there is one network user (OR = 2.04, 95% CI = 1.42, 2.94); approximately tripled for two (OR = 2.99, 95% CI = 1.83, 4.89) and three users (OR = 2.82, 95% CI = 1.32, 6.00); more than 4.5 times higher for four users (OR = 4.68, 95% CI = 1.74, 12.58); and approximately 3.5 times higher for five users (OR = 3.46, 95% CI = 1.10, 10.88). This indicates strong clustering of contraceptive users in families and among the close ties of social networks.

Independent of all these effects, community-level predictors are important in both models (figure 2, CL model), though in different ways. A 1 s.d. increase in mean education in the community has the same effect on the odds of contraceptive use (36% increased odds; OR = 1.36, 95% CI = 1.13, 1.63) as a 1 s.d. increase in individual education (OR = 1.36, 95% CI = 1.17, 1.58). This is consistent with our previous work, which showed that community education has independent effects on fertility decline in this population [36]. However, community-level education is not associated with artificial method use (and neither is individual-level education). Surprisingly, the presence of a health centre in the community is associated with *reduced* odds of using any contraceptive methods (OR = 0.69, 95% CI = 0.50, 0.96) and is not associated with artificial method use. Community-level religiosity is not associated with reductions in contraceptive use in general, only with reductions in the odds of artificial contraceptive use. A 1 s.d. increase in mean religiosity in the community is associated with 19% lower odds of using artificial methods (OR = 0.81, 95% CI = 0.68, 0.96), over and above the strong effect of individual religiosity.

### (c) Model comparisons

When pitted directly against each other (i.e. assuming different classes are mutually exclusive) and controlling for life-history characteristics, the social network and kin effects (SN) model receives the most support for both contraceptive outcomes, taking 100% of the available Akaike weight (*ω*_i_), interpreted as the probability that the model best approximates the data at hand [55] (table 2). The contraceptive status of other people is thus substantially better at accounting for the variation in women's contraceptive use than are the other classes of predictors.

**Table 2.** Comparison of each model against the others, controlling for life-history predictors (LH). SES, socioeconomic predictors; IS, individual sociocultural predictors; SN, social network and kin effects; CL, community-level effects; *k*, estimated effective no. of parameters; logLik, logLikelihood; DIC, deviance information criterion; ΔDIC, DIC difference from top model; *ω*_i_, Akaike weight.

model	*k*	logLik	DIC	ΔDIC	*ω*_i_
(i) any methods					
LH + SN	20	−955.53	1911.1	0.00	1.00
LH + SES	15	−1006.54	2013.1	102.03	0.00
LH + IS	13	−1012.09	2024.2	113.13	0.00
LH + CL	14	−1029.19	2058.4	147.33	0.00
LH	10	−1042.06	2084.1	173.06	0.00
(ii) artificial methods					
LH + SN	20	−843.07	1686.1	0.00	1.00
LH + IS	13	−875.41	1750.8	64.69	0.00
LH + SES	15	−887.80	1775.6	89.47	0.00
LH + CL	14	−905.58	1811.2	125.03	0.00
LH	10	−916.96	1833.9	147.80	0.00

When we compare all possible combinations of the different model classes (32 different models, including a null model), the global models best account for the data in both cases, again taking 100% of the Akaike weight (table 3). Analyses containing multiple levels of analysis and sources of influence are therefore better able to capture variation in contraceptive use than are individual or social network analyses alone. These model comparison outcomes do not differ when using different information criteria (AICc; electronic supplementary material, tables S9 and S10) or when the *a priori* models are reduced to their most parsimonious forms prior to comparison (electronic supplementary material, tables S11 and S12).

**Table 3.** Comparison of every combination of models (32 possibilities; only the top five models are shown). LH, life-history predictors; SES, socioeconomic predictors; IS, individual sociocultural predictors; SN, social network and kin effects; CL, community-level effects; *k*, estimated effective number of parameters; logLik, logLikelihood; DIC, deviance information criterion; ΔDIC, DIC difference from top model; *ω*_i_, Akaike weight.

model	*k*	logLik	DIC	ΔDIC	*ω*_i_
(i) any methods					
LH + SES + IS + SN + CL	32	−915.59	1831.2	0.00	1.00
LH + SES + IS + SN	28	−924.64	1849.3	18.11	0.00
LH + SES + SN + CL	29	−925.51	1851.0	19.84	0.00
LH + IS + SN + CL	27	−926.28	1852.6	21.38	0.00
LH + SES + SN	25	−935.16	1870.3	39.14	0.00
(ii) artificial methods					
LH + SES + IS + SN + CL	32	−795.57	1591.1	0.00	1.00
LH + SES + IS + SN	28	−803.02	1606.0	14.92	0.00
LH + IS + SN + CL	27	−803.36	1606.7	15.58	0.00
LH + IS + SN	23	−811.79	1623.6	32.46	0.00
LH + SES + SN + CL	29	−818.78	1637.6	46.43	0.00

## 3. Discussion

Our study simultaneously integrates individual-, social network- and community-level factors as combined predictors of contraceptive use, and is to our knowledge the first to systematically compare these multilevel effects against each other and on different contraceptive outcomes. Interpersonal and sociocultural factors, in particular the contraceptive behaviour of friends and family, have the largest and most consistent effects on the probability of contraceptive use, followed by a woman's religiosity, with routinely used socioeconomic and life-history predictors being either inconsistent or unimportant predictors. However, the religiosity and education of women's neighbours appears to independently constrain and facilitate the use of different kinds of contraception, suggesting that community-level dynamics are important additional drivers of contraceptive behaviour.

Fertility reduction in this population has been driven mainly by the use of NFP, with artificial methods not associated with more dramatic fertility reductions (table 1). Decisions to reduce and/or control fertility in this population are therefore just as well proxied by ‘natural’ as by ‘artificial’ contraceptive methods. But because our results show that different individual- and community-level predictors matter for different method types, it follows that studies focusing only on modern contraceptive uptake could generate misleading interpretations about the social transmission of low-fertility norms. By taking a comparative approach to understanding different sources of influence, our results generate four insights into the cultural dynamics that may partly drive contraceptive behaviour, and which may be independent of both the economic opportunities women are exposed to and the life-history stages they find themselves in.

First, strongly correlated contraceptive behaviour among friends and families may indicate a frequency-dependent probability of contraceptive use, whereby women adopt the contraceptive behaviour (and by proxy the reproductive norms) of their preferred social models as they become more frequent in their social networks. This is a pattern that is predicted by a range of evolutionary models [32,47,49,59]. Our results differ from two seminal demographic studies which demonstrated (1) nonlinear, threshold effects of ego-network partners on women's contraceptive use, interpreted as evidence for purposeful information-seeking over social transmission [50], and (2) that social transmission is mediated by the density of social networks, which can constrain or facilitate contraceptive diffusion [52]. Our results instead suggest a broadly linear increase in the odds of contraceptive use with additional users in the network that is more consistent with frequency-dependent adoption, and controlling for network density does not affect our results (electronic supplementary material, table S7). Our analysis does not allow us to explicitly distinguish clustering from contagion. However, we can rule out homophily along educational lines as a major driver of our results, because controlling for mean education of network partners does not affect our outcomes (electronic supplementary material, table S8) and because neither individual- nor community-level education were important predictors of artificial contraceptive use.

Second, our results suggest that horizontal (peer-to-peer) and/or oblique (non-parents of the parental generation) social transmission among kin and close network ties may be more important than vertical transmission of contraceptive behaviour from mothers to daughters in this population. This is consistent with the hypothesis that relatively rapid ecological change de-emphasizes the importance of vertical transmission. Theoretically, mothers should have the most accurate information about the lifetime costs and benefits to controlling fertility, having typically finished reproducing by the time their daughters begin, by which point their pay-offs are observable. Intergenerational correlations in fertility, which become more pronounced as demographic transitions progress, imply strong cultural transmission of reproductive behaviour, such as age at marriage and first birth, between mothers and daughters [60]. The lack of a mother effect on the odds of using artificial methods here might be due to differential availability for mothers and daughters, but this is not the case for natural methods, which have been used in all age cohorts (electronic supplementary material, table S3). Instead, maternal reproductive pay-offs may be outdated, as the costs and benefits of controlling fertility are changing in the course of the subsistence transition that is ongoing [36,56]. Reproductive decisions are inherently future-oriented, and this alone should increase the salience of contraceptive information from peers, accelerating rates of horizontal and oblique transmission in transitioning populations, even in the absence of substantial educational changes. A deeper understanding of these dynamics will require more formal modelling and multilevel analysis.

Third, our results show that, independent of a woman's own religiosity, her probability of artificial contraceptive use is influenced by how religious her neighbours are, but this is not the case for other methods. Even a relatively non-religious woman is therefore less likely to use artificial contraceptives simply because others in her community are religious. This contextual effect is not simply due to opportunities to interact with and access the Church. Community-level religiosity may reinforce a reluctance to use artificial contraceptives, promoting greater homogeneity in contraceptive practices. This is broadly consistent with conformist transmission [31,32] and with evidence that women conform to the reproductive expectations they perceive to be normative in their community [28].

Fourth, cultural evolutionary models predict that the ‘background’ distribution of cultural traits in a group—in particular average education—can facilitate the transmission of secondary cultural traits, such as small family-size preferences or contraceptive behaviour [30,35]. This cultural niche-constructing process can theoretically accelerate contraceptive diffusion enough that groups can overcome the effects of conformity [35]. Our results are generally consistent with this interpretation, but comparison of different method types is needed to understand these dynamics. Community-level education appears to accelerate generalized contraceptive uptake more than community-level religiosity slows it down, but the opposite is true for artificial methods: community-level religiosity appears to limit the use of these more controversial methods more strongly than community-level education accelerates it.

It is important to disentangle network- from community-level effects because personal networks represent private spaces of social interaction within which broader community-level norms may be violable. While publicly conforming to ‘consensus' reproductive norms, even actively promoting them, women can learn about and experiment with new contraceptive methods in the safety of their private networks. A critical mass of contraceptive users is easier to achieve within the network than the community [51], which can explain why communities that ostensibly promote traditional norms nonetheless end up with substantial proportions of contraceptive users [16] (see also [28]). Our finding that neither exposure to social media nor having a health centre in the community positively influenced artificial contraceptive use further suggests that formal information sources may be less important than are informal sources and that information diffusion is not equivalent to behaviour change. Development interventions increasingly recognize that the most influential sources of information are the close ties of social networks, and targeting specific network partners as ‘opinion leaders' has been a successful strategy in disseminating contraceptive behaviour [12,41]. Our results further suggest that, where the primary interest is in reducing fertility (rather than tackling HIV prevalence), a much wider range of more culturally acceptable methods should be targeted and/or information about effective use of NFP prioritized.

In our study communities, economic change is creating new opportunities for education, social mobility, wealth and status creation [61]. Social networks are becoming more educated, potentially opening up new channels of social interaction [36], which can rapidly spread information about the costs and benefits to fertility limitation. Our study is unusual in the range of methods, information and socioeconomic options available to women. Future research should aim to examine a wider range of contraceptive methods to shed further light on the dynamics of cultural transmission, which may differentially account for the prevalence of particular method types. Unless the economic conditions in a particular context are conducive to fertility decline, and the pace of change sufficiently rapid to generate widespread social learning, it may be difficult to uncover positive evidence of social transmission of modern contraceptive uptake [7,8], because contraceptives are strategically used to control mortality and manage the rate of reproduction, without compromising high-fertility norms [7,53]. Lack of diffusion may also indicate widespread conformity to high-fertility norms. By exclusively focusing on particular methods when others may do just as well as proxies, important questions about the mechanisms of significant fertility limitation may be obscured. The results presented here show that across multiple method types, social-structural and cultural dynamics are centrally important influences on contraceptive use.

## 4. Material and methods

Data were collected between 2009 and 2010 as part of an anthropological–demographic project. All variables are described in the electronic supplementary material. Education and religiosity were group-mean centred so that community means did not introduce confounding colinearity. We exclude network density from our SN model, as this removes women with only one or no network partners. Electronic supplementary material analysis reveals that model outcomes do not differ when including (i) network density (electronic supplementary material, table S7) or (ii) mean network education (also reducing sample size, electronic supplementary material, table S8). All data were analysed in R v. 3.1.1 [62].

## Supplementary Material

Electronic Supplementary Material

## Data accessibility

All data used in the paper are available on request from Heidi Colleran at heidi.colleran@iast.fr.
