# Supplementary material for: Social network- and community-level influences on contraceptive use: evidence from rural Poland
Source: Proc Biol Sci. 2015 May 22;282(1807):20150398. doi: 10.1098/rspb.2015.0398 (PMC4424654; doi:10.1098/rspb.2015.0398)
Supplement: Electronic Supplementary Material [file rspb20150398supp1.pdf]

Electronic Supplementary Materials

"*Social network and community level influences on contraceptive use: evidence from rural Poland*"

Heidi Colleran and Ruth Mace

Contents:

Supplementary Discussion

Supplementary Tables S1-S12

Supplementary References

Supplementary discussion

1. Data collection

Our sample of communities (21 villages and one town) was randomly drawn from four neighbouring municipalities containing a total of 34 potential study populations. All 22 of our sampled communities agreed to participate. We did not have access to a list of names or a map of households, so within each community every third house was sampled, with every adult woman (≥18 years) present in the house invited to take part in the survey. This strategy was further stratified in the town, by randomly selecting streets from a list obtained from the local government and approaching every third house/apartment on that particular street. We returned to houses that were unoccupied at the time of selection on up to three occasions. All consenting adult women were interviewed. All eligible women who declined to take part were noted as non-responders, as were women ineligible to take part due to age or illness. 52.4% to 89.4% of respondents who were approached in any village agreed to an interview, and the average response rate for the entire sample was 75% (total interviews ÷ [total responders + non-responders]). Our sampling strategy means that important variables such as age are approximately normally distributed in all groups. The sample should not be considered representative of the country as a whole, but of the wider rural population of this particular region.

As part of a wider survey interview, respondents were asked about their current and past use of 15 different contraceptive methods. These were then classified as either natural or artificial methods. Natural methods include calendar and sympto-thermal methods, withdrawal/*coitus interruptus* and periodic abstinence. Artificial methods include condoms, birth control pills, IUDs, post-coital or emergency contraception ('morning-after' pills), spermicides, sterilisation, hormonal implants and patches, and diaphragms (Table 1).

Respondents were asked to name up to five female social network partners with whom they could talk about "important personal matters". These alters could be kin or non-kin, geographically proximate or living far away, and respondents were encouraged to nominate only those most important to them rather than being forced to name five people. Respondents reported whether each nominated alter in their social network had ever used a natural and/or artificial contraceptive method, and whether a member of their family had ever used any methods of contraception. Where respondents did not know the contraceptive status of their kin or friends, or where they did not give information about them, it was assumed that these individuals had not used any method of contraception. There were almost no cases where a known dyad (i.e. two respondents who nominated each other) gave inconsistent information about each other's contraceptive use. Nonetheless, the data are likely to underestimate the number of kin and alters using contraceptives, and as a result our results should be considered conservative measures of the influence of alters on respondents' contraceptive status.

Table S1 details the observed frequency of kin and social network partners using either any or artificial methods of contraception.

**Number of respondents whose kin/friends used contraception**	Any method	Artificial method
mother used contraception	266	23
sister(s) used contraception	382	115
other kin used contraception	287	318
0 alters used contraception	1136	1436
1 alter used contraception	216	204
2 alters used contraception	204	109
3 alters used contraception	128	51
4 alters used contraception	96	32
5 alters used contraception	74	22

Note that 118 respondents did not nominate any friends

Table S1 Description of the number of respondents who had kin and/or social network partners who used either any method or an artificial method of contraception.

The personal networks reported here are fairly well mixed between kin and non-kin, are characterized by long-term friendships with a high degree of interpersonal contact and are located in a relatively limited geographical area. Table S2 gives an overview of the main characteristics of these personal networks. On average, women have known their network partners for over 23 years (s.d. = 13.57), indicating that the data capture long-term rather than transient personal ties. Approximately 47% of all network partners were kin, with the remaining ~52% being non-kin. 49% of all alters originated in the same village that ego currently lives in, with 52% currently resident in the same village. Most of the remaining alters (~40%) were currently living in the local villages and towns, with fewer than 7% of all alters living in a city or foreign country. Frequency of both face-to-face and telephone/email contact was high among network partners, with over 43% of friends being physically visited and over 66% being contacted by other means (mainly telephone) on a daily basis.

Variable	mean	s.d.	range
Number of friends (all respondents)	2.92	1.46	0 to 5
Number of friends (nominators only)	3.11	1.30	1 to 5
Friendship duration (years)	23.18	13.57	0.67 to 78
Number of kin	1.37	1.33	0 to 5
Number non-kin	1.55	1.32	0 to 5
Origin of friends	percentage		
From same village as ego	49.47%		
From another local village	36.39%		
From another town	10.23%		
From a city	3.72%		
From another country	0.19%		
Current location of friends			
Resident in same village as ego	52.70%		
Resident in other village	28.54%		
Resident in other town	11.48%		
Resident in a city	5.67%		
Resident in another country	1.62%		
Frequency of face-to-face contact			
Daily	43.69%		
Weekly	25.12%		
Few times a month	14.44%		
Once a month	8.59%		
Few times a year or less	8.16%		
Frequency of phone/email contact			
Daily	66.81%		
Weekly	22.65%		
Few times a month	6.69%		
Once a month	2.82%		
Few times a year or less	1.02%		

Table S2 Description of social networks

	**age at first contraceptive use**								
	(a) natural			(b) artificial			(c) any method		
**Cohort**	mean	s.d.	n	mean	s.d.	n	mean	s.d.	n
Born before 1930	22.25	3.10	4	.	.	.	22.25	3.10	4
Born before 1940	25.47	5.29	38	22.00	4.24	2	25.47	5.29	38
Born before 1950	23.75	5.06	96	29.10	9.23	10	23.70	4.89	98
Born before 1960	23.13	3.36	176	26.77	6.72	26	23.20	3.44	184
Born before 1970	22.69	3.97	243	25.79	4.96	80	22.95	4.18	258
Born before 1980	22.46	3.36	257	23.73	4.13	171	22.60	3.51	309
Born before 1992	19.82	2.49	246	20.03	2.41	231	19.74	2.47	328
**Grand mean (women born before 1980)**	23.29	4.02	814	25.48	5.86	289	23.36	4.07	891

Table S3 Age at first contraceptive use by cohort and type of contraception. Contraceptives of some sort have been used in all age cohorts.

Table S4 gives a breakdown of completed fertility differences between users and non-users of the most popular contraceptive methods. Significant differences in completed fertility are mainly driven by use of sympto-thermal methods of contraception. Although the trend is for artificial contraceptive users to have fewer children than non-users of these particular methods, the differences are not statistically significant.

Completed fertility by selected contraceptive method (45+)

	non-users			users					
Method	mean	sd	n	mean	sd	n	t-value	p-value	
Natural									
calendar	3.92	2.27	557	3.65	1.93	350	1.91	0.057	
withdrawal	3.83	2.23	761	3.75	1.63	146	0.46	0.649	
sympto-thermal	3.89	2.21	785	3.35	1.67	122	3.12	0.002	**
abstinence	3.82	2.15	861	3.61	2.10	46	0.68	0.501	
Artificial									
condom	3.83	2.18	851	3.13	1.67	56	1.75	0.101	
pill	3.83	2.16	892	3.13	1.51	15	1.75	0.101	
IUD	3.82	2.15	904	3.33	1.15	3	0.72	0.545	

Welch t-tests assuming unequal variances $p < 0.05$*, $p < 0.01$**, $p < 0.001$***

Table S4 Completed fertility of post-reproductive aged women (45+) broken down by method type.

2. Variables used in the analysis

We used a combination of direct and composite measures in our analyses (see [1] for further details). Education, material wealth, market integration and farming wealth were standardised to have a mean of 0 and s.d. of ~1. Education was measured as the highest level of formal education obtained by the respondent. This ranged from 1 = none/some primary (n = 21), 2 = full primary (n = 317), 3 = vocational (n = 523), 4 = secondary (n = 806) to 5 = tertiary (n = 305).

Household material wealth is a weighted linear combination of six variables, created using Principal Component Analysis (PCA): mean household income (measured across all adults in the household in brackets ranging from <600 Polish Zlotys [PLN] per month, to >2,500 PLN per month), ownership of a computer, an internet connection, a satellite TV, a car, as well as the number of habitable rooms in the house. This measure captures both income and assets, and covers the whole household rather than simply the parents or the husband of the respondent. The variables used to construct this measure show good inter-item scalability (Cronbachs α= 0.71), and the first Principal Component accounted for 42% of the variance in the original items.

Household market integration is a weighted composite measure capturing the extent to which the household is dependent on wage-labour versus farming income. For every householder, occupation (ranging from student to farmer to full-time employed), occupational status (if employed) and an indicator of employment history (ever employed) were weighted to provide a cumulative 'market integration' score. We took the household mean as the measure of

overall market integration. The respondent is not included in this measure to avoid confounding co-linearity with the other measures, specifically individual education. We control for farming status (resident in a farming household at the time of the survey or not; n 'farmers' = 1,239, n 'non-farmers' = 733), experience of under-five mortality (n 'yes' = 65, n 'no' = 1,907) and age of the respondent (mean =44, s.d. = 17.83, range = 18-91). We also included age squared and age cubed to account for the non-linear relationship between fertility and age across the lifespan. 23 women who were not currently resident in any of the sampled communities were excluded from the analysis, leaving a total sample of 1,972.

Degree of religiosity was assessed using three variables; frequency of attendance at Mass, a four-point scale ranging from 'never' to 'more than once a week', self-identified religiosity, based on the question "*would you say you were a religious person*?" and coded as a four-point scale ranging from 'not religious at all' to 'very religious', and self-reported importance of religion in the person's life, based on the question "*how important would you say religion is in your daily life*?", a five-point scale ranging from 'not at all important' to 'very important'. These variables were combined into a single dimension using principal component analysis. The first component explains a large proportion (56%) of the variance in the original data and the variables show good inter-item scalability (Crohnbach's $\alpha = 0.61$). Our '*religiosity*' score has a mean of 0 and a s.d. of 1.

'*Social media exposure*' is a principal component, made up of three variables indicating personal ownership of a mobile phone and regular use of computers and the internet (i.e. independent of ownership of a computer). Again the variables show good inter-item scalability (Cronbach's $\alpha = 0.83$) and the dimension explains a large proportion of the variation in the original data (~75%). The score has a mean of 0 and a s.d. of 1.

Table S5 gives a correlation matrix of the community level predictors considered in the multilevel models. The table shows that there is low-to-moderate inter-correlation between the measures. There is a moderate correlation between the presence of a church and a health centre (Pearson's R = 0.49, $p < 0.001$), most likely due to some villages having more amenities overall than others. Note that average religiosity is not highly correlated with the presence of a church in the village.

We control for the presence of a health centre and/or a church in the community in our models because these are proxies both for access to contraceptives and for information on the social, financial and physiological costs and benefits to using artificial methods. This allows us to better estimate whether the effects of average religiosity and education are the result of unobserved aspects of shared environment or cultural norms. Twelve of the 22 study communities contain a church and four communities contain a public health centre, with the main hospital (where most children are born) being in the town. We group-mean centred individual education and religiosity to allow the reintroduction of the uncorrelated group means (average community education and religiosity) later in the analysis.

Correlation matrix of community level variables considered in the analysis

	Mean religiosity		Church		Health center	
Church in the community	0.11	***	.		.	
Health center in community	0.18	***	0.49	***	.	
Mean educational capital	-0.48	***	0.11	***	0.18	***

Pearson's R. $p < 0.05$*, $p < 0.005$**, $p < 0.001$***

Table S5 Correlation matrix of the group level variables considered in the analysis. All measures show low-to-moderate intercorrelation.

3. Statistical analysis

The multilevel logistic regression models estimate the change in the log-odds of having ever used either method based on a unit change in the predictors (Tables S6-S8). All analyses were run in the R environment, version 3.1.1 [2], using the 'arm' [3], 'lme4' [4], 'blme' [5] and 'ggplot2' [6] packages. Model comparison was carried out using the 'MuMIn' package [7].

To avoid boundary problems associated with small variance parameters and a small sample of groups, including incorrect or underestimated uncertainty in the model parameters and covariance matrices, we used maximum penalized likelihood (MPL)[8,9], with the Laplace approximation, to estimate our models. MPL uses 'weakly informative' or 'diffuse' priors to obtain Bayesian modal estimates on the parameters in the model. This ensures that the variance component estimates remain off the boundary of the feasible parameter space (zero), but with weak enough priors so that inferences remain consistent with the data. This method extends standard multilevel modelling techniques without requiring simulation as in fully Bayesian analysis (which obtains posterior mean estimates) and outperforms standard maximum likelihood methods.

We did not include controls for age at first birth or length of first inter-birth interval as this results in the exclusion of women who have not yet had any children. We included unmarried women in the analysis because a sizeable proportion of unmarried women have used artificial methods of contraception (n = 125, 24% of artificial contraceptive users). Our models included only those predictors that have been shown to be salient in previous demographic and evolutionary research on contraceptive uptake.

(i) Any methods

Parameter	β	SE	z value	Pr(>|z|)		OR	lower CI	upper CI
Intercept	-3.54	1.27	-2.79	0.01	**	0.03	0.00	0.35
Life history variables								
Parity 0 (ref = no)	-0.82	0.33	-2.48	0.01	*	0.44	0.23	0.84
Parity 1 to 2 (ref = no)	0.22	0.20	1.11	0.27		1.25	0.84	1.85
Parity 3 to 4 (ref = no)	0.31	0.17	1.77	0.08		1.36	0.97	1.91
Parity 5 and above (reference category)	.	.	.	.		.	.	.
Age	0.23	0.09	2.63	0.01	**	1.26	1.06	1.49
Age^2	0.00	0.00	-2.40	0.02	*	1.00	0.99	1.00
Age^3	0.00	0.00	1.85	0.06	.	1.00	1.00	1.00
Marital status (ref = unmarried)	1.09	0.28	3.88	0.00	***	2.97	1.71	5.14
Experience of under-5 mortality (ref = no)	-0.56	0.33	-1.67	0.09	.	0.57	0.30	1.10
Socioeconomic variables								
Farmer (ref = no)	-0.11	0.15	-0.72	0.47		0.90	0.66	1.21
Relative education	0.31	0.08	3.95	0.00	***	1.36	1.17	1.58
Household material resources	0.03	0.07	0.38	0.70		1.03	0.90	1.18
Household market integration	0.09	0.08	1.12	0.26		1.09	0.94	1.27
Household farming wealth	0.09	0.07	1.23	0.22		1.10	0.95	1.27
Individual sociocultural predictors								
Exposure to social media	0.31	0.10	3.15	0.00	**	1.36	1.12	1.65
Ever migrated (ref = no)	0.26	0.12	2.13	0.03	*	1.30	1.02	1.65
Relative religiosity	-0.13	0.06	-2.02	0.04	*	0.88	0.77	1.00
Social network and kin variables								
Mother used any method (ref =no)	0.29	0.28	1.04	0.30		1.34	0.77	2.31
Any sister used any method (ref = no)	0.63	0.26	2.41	0.02	*	1.88	1.12	3.15
Other kin used any method(ref = no)	0.39	0.20	1.98	0.05	*	1.48	1.00	2.18
Number social network alters used any method (ref = none)	.	.	.	.		.	.	.
1 alter used any method	0.54	0.20	2.67	0.01	**	1.72	1.16	2.56
2 alters used any method	1.13	0.26	4.43	0.00	***	3.11	1.88	5.14
3 alters used any method	0.91	0.33	2.76	0.01	**	2.49	1.30	4.76
4 alters used any method	0.61	0.37	1.64	0.10		1.84	0.89	3.81
5 alters used any method	2.72	0.88	3.09	0.00	**	15.14	2.70	84.82
Proportion kin in the network	-0.07	0.16	-0.46	0.64		0.93	0.68	1.27
Social network size	0.02	0.04	0.57	0.57		1.02	0.94	1.12
Community level variables								
Church in the village (ref = no)	-0.06	0.17	-0.34	0.73		0.95	0.68	1.31
Health center in the village (ref = no)	-0.37	0.17	-2.19	0.03	*	0.69	0.50	0.96
Mean religiosity	0.01	0.08	0.13	0.90		1.01	0.86	1.18
Mean education	0.31	0.09	3.30	0.00	***	1.36	1.13	1.63
Variance component								
Between-community variance	0.008							
Model summary and fit								
Sample size	1972							
logLikelihod	-915.6							
DIC	1831.2							

$p < 0.05$*, $p < 0.005$**, $p < 0.001$***

(ii) Artificial methods

Parameter	β	SE	z value	Pr(>|z|)		OR	lower CI	upper CI
Intercept	-6.05	1.51	-4.01	0.00	***	0.00	0.00	0.05
Life history variables								
Parity 0 (ref = no)	-0.79	0.40	-1.99	0.05	*	0.45	0.21	0.99
Parity 1 to 2 (ref = no)	-0.13	0.28	-0.48	0.63		0.87	0.51	1.51
Parity 3 to 4 (ref = no)	0.01	0.26	0.04	0.97		1.01	0.60	1.69
Parity 5 and above (reference category)	.	.	.	.		.	.	.
Age	0.43	0.11	3.89	0.00	***	1.54	1.24	1.91
Age^2	-0.01	0.00	-4.20	0.00	***	0.99	0.98	0.99
Age^3	0.00	0.00	3.86	0.00	***	1.00	1.00	1.00
Marital status (ref = unmarried)	0.13	0.30	0.45	0.65		1.14	0.64	2.04
Experience of under-5 mortality (ref = no)	-0.22	0.49	-0.44	0.66		0.81	0.31	2.10
Socioeconomic variables								
Farmer (ref = no)	-0.01	0.16	-0.04	0.97		0.99	0.73	1.36
Relative education	0.14	0.09	1.57	0.12		1.15	0.97	1.38
Household material resources	0.24	0.10	2.32	0.02	*	1.27	1.04	1.56
Household market integration	0.06	0.08	0.71	0.48		1.06	0.90	1.25
Household farming wealth	-0.12	0.08	-1.42	0.16		0.89	0.76	1.05
Individual sociocultural predictors								
Exposure to social media	0.11	0.12	0.93	0.35		1.12	0.88	1.41
Ever migrated (ref = no)	0.22	0.14	1.65	0.10	.	1.25	0.96	1.63
Relative religiosity	-0.46	0.07	-6.35	0.00	***	0.63	0.55	0.73
Social network and kin variables								
Mother used artificial method (ref =no)	0.28	0.55	0.50	0.61		1.32	0.45	3.85
Any sister used artificial method (ref = no)	0.59	0.29	2.05	0.04	*	1.81	1.03	3.17
Other kin used artificial method(ref = no)	0.57	0.16	3.59	0.00	***	1.78	1.30	2.43
Number social network alters used artificial method (ref = none)	.	.	.	.		.	.	.
1 alter used artificial method	0.71	0.19	3.82	0.00	***	2.04	1.42	2.94
2 alters used artificial method	1.10	0.25	4.37	0.00	***	2.99	1.83	4.89
3 alters used artificial method	1.04	0.39	2.69	0.01	**	2.82	1.32	6.00
4 alters used artificial method	1.54	0.50	3.06	0.00	**	4.68	1.74	12.58
5 alters used artificial method	1.24	0.59	2.12	0.03	*	3.46	1.10	10.88
Proportion kin in the network	0.02	0.18	0.10	0.92		1.02	0.71	1.46
Social network size	-0.02	0.05	-0.41	0.68		0.98	0.89	1.08
Community level variables								
Church in the village (ref = no)	0.13	0.18	0.71	0.48		1.14	0.80	1.62
Health center in the village (ref = no)	0.05	0.18	0.29	0.78		1.05	0.74	1.50
Mean religiosity	-0.21	0.09	-2.47	0.01	*	0.81	0.68	0.96
Mean education	0.07	0.10	0.72	0.47		1.08	0.88	1.31
Variance component								
Between-community variance	0.011							
Model summary and fit								
Sample size	1972							
logLikelihod	-795.6							
DIC	1591.1							

$p < 0.05$*, $p < 0.005$**, $p < 0.001$***

Table S6 Global multilevel logistic regression models predicting the odds of (i) any method and (ii) artificial methods of contraception

(i) Any methods

Parameter	Estimate	Std. Error	z value	Pr(>|z|)	
Intercept	-2.99	1.58	-1.90	0.058	.
Life history variables					
Parity 0 (ref = no)	-1.12	0.38	-2.94	0.003	**
Parity 1 to 2 (ref = no)	-0.01	0.24	-0.06	0.953	
Parity 3 to 4 (ref = no)	0.19	0.21	0.91	0.361	
Parity 5 and above (reference category)					
Age	0.24	0.11	2.23	0.026	*
Age^2	0.00	0.00	-1.92	0.055	.
Age^3	0.00	0.00	1.36	0.172	
Marital status (ref = unmarried)	0.84	0.33	2.57	0.010	*
Experience of under-5 mortality (ref = no)	-0.32	0.40	-0.80	0.425	
Socioeconomic variables					
Farmer (ref = no)	-0.20	0.18	-1.13	0.259	
Relative education	0.34	0.09	3.78	0.000	***
Household material resources	0.07	0.08	0.88	0.377	
Household market integration	0.15	0.16	0.91	0.361	
Household farming wealth	0.16	0.09	1.88	0.061	.
Individual sociocultural predictors					
Exposure to social media	0.21	0.12	1.78	0.075	.
Ever migrated (ref = no)	0.37	0.14	2.59	0.010	**
Relative religiosity	-0.15	0.08	-1.99	0.047	*
Social network and kin variables					
Mother used any method (ref =no)	0.23	0.29	0.78	0.437	
Any sister used any method (ref = no)	0.65	0.27	2.38	0.017	*
Other kin used any method(ref = no)	0.35	0.22	1.60	0.110	
Number social network alters used any method (ref = none)					
1 alter used any method	0.47	0.24	2.01	0.045	*
2 alters used any method	1.14	0.28	4.05	0.000	***
3 alters used any method	0.90	0.34	2.63	0.008	**
4 alters used any method	0.66	0.38	1.74	0.082	.
5 alters used any method	2.79	0.89	3.14	0.002	**
Proportion kin in the network	-0.27	0.20	-1.34	0.181	
Social network size	-0.05	0.07	-0.72	0.474	
Network density	-0.09	0.32	-0.27	0.790	
Community level variables					
Church in the village (ref = no)	0.02	0.20	0.11	0.911	
Health center in the village (ref = no)	-0.46	0.20	-2.31	0.021	*
Mean religiosity	0.03	0.09	0.35	0.728	
Mean education	0.36	0.11	3.28	0.001	**
Variance component					
Between-community variance	0.015				
Model summary and fit					
Sample size	1516				
logLikelihod	-693.00				
DIC	1385.95				

$p < 0.05$*, $p < 0.005$**, $p < 0.001$***

(ii) Artificial methods

Parameter	Estimate	Std. Error	z value	Pr(>|z|)	
Intercept	-6.57	1.89	-3.48	0.001	***
Life history variables					
Parity 0 (ref = no)	-0.98	0.45	-2.18	0.030	*
Parity 1 to 2 (ref = no)	-0.21	0.32	-0.67	0.506	
Parity 3 to 4 (ref = no)	-0.24	0.30	-0.80	0.423	
Parity 5 and above (reference category)					
Age	0.51	0.14	3.63	0.000	***
Age^2	-0.01	0.00	-3.72	0.000	***
Age^3	0.00	0.00	3.23	0.001	**
Marital status (ref = unmarried)	-0.04	0.34	-0.13	0.899	
Experience of under-5 mortality (ref = no)	0.01	0.60	0.01	0.990	
Socioeconomic variables					
Farmer (ref = no)	-0.06	0.18	-0.35	0.725	
Relative education	0.09	0.10	0.86	0.388	
Household material resources	0.33	0.12	2.69	0.007	**
Household market integration	0.10	0.17	0.60	0.546	
Household farming wealth	-0.13	0.09	-1.39	0.164	
Individual sociocultural predictors					
Exposure to social media	-0.05	0.14	-0.35	0.724	
Ever migrated (ref = no)	0.20	0.15	1.29	0.197	
Relative religiosity	-0.46	0.08	-5.54	0.000	***
Social network and kin variables					
Mother used artificial method (ref =no)	0.33	0.56	0.59	0.554	
Any sister used artificial method (ref = no)	0.54	0.30	1.79	0.074	.
Other kin used artificial method(ref = no)	0.56	0.17	3.19	0.001	**
Number social network alters used artificial method (ref = none)					
1 alter used artificial method	0.71	0.22	3.28	0.001	**
2 alters used artificial method	1.10	0.27	4.05	0.000	***
3 alters used artificial method	1.00	0.39	2.54	0.011	*
4 alters used artificial method	1.59	0.51	3.10	0.002	**
5 alters used artificial method	1.48	0.62	2.40	0.016	*
Proportion kin in the network	-0.13	0.22	-0.57	0.567	
Social network size	-0.02	0.07	-0.24	0.807	
Network density	-0.07	0.31	-0.24	0.812	
Community level variables					
Church in the village (ref = no)	0.13	0.21	0.64	0.522	
Health center in the village (ref = no)	0.11	0.20	0.56	0.573	
Mean religiosity	-0.22	0.10	-2.25	0.025	*
Mean education	0.03	0.12	0.25	0.805	
Variance component					
Between-community variance	0.015				
Model summary and fit					
Sample size	1516				
logLikelihod	-623.20				
DIC	1246.42				

$p < 0.05$*, $p < 0.005$**, $p < 0.001$***

Table S7 Global multilevel logistic regression models predicting the odds of (i) any method and (ii) artificial methods of contraception, including network density as a control.

(i) Any methods

Parameter	Estimate	Std. Error	z value	Pr(>|z|)	
Intercept	-3.83	1.35	-2.85	0.004	**
Life history variables					
Parity 0 (ref = no)	-0.98	0.35	-2.78	0.005	**
Parity 1 to 2 (ref = no)	0.10	0.22	0.43	0.665	
Parity 3 to 4 (ref = no)	0.26	0.19	1.37	0.170	
Parity 5 and above (reference category)					
Age	0.29	0.09	3.14	0.002	**
Age^2	-0.01	0.00	-2.89	0.004	**
Age^3	0.00	0.00	2.33	0.020	*
Marital status (ref = unmarried)	1.01	0.30	3.35	0.001	***
Experience of under-5 mortality (ref = no)	-0.65	0.36	-1.78	0.075	.
Socioeconomic variables					
Farmer (ref = no)	-0.16	0.16	-0.96	0.336	
Relative education	0.29	0.09	3.25	0.001	**
Household material resources	0.06	0.08	0.75	0.456	
Household market integration	0.05	0.16	0.29	0.771	
Household farming wealth	0.11	0.08	1.43	0.153	
Individual sociocultural predictors					
Exposure to social media	0.33	0.11	3.11	0.002	**
Ever migrated (ref = no)	0.27	0.13	2.06	0.040	*
Relative religiosity	-0.19	0.07	-2.67	0.008	**
Social network and kin variables					
Mother used any method (ref =no)	0.35	0.29	1.21	0.228	
Any sister used any method (ref = no)	0.57	0.27	2.12	0.034	*
Other kin used any method(ref = no)	0.34	0.20	1.68	0.094	.
Number social network alters used any method (ref = none)					
1 alter used any method	0.47	0.21	2.21	0.027	*
2 alters used any method	1.11	0.27	4.17	0.000	***
3 alters used any method	0.96	0.34	2.86	0.004	**
4 alters used any method	0.70	0.38	1.85	0.064	.
5 alters used any method	2.88	0.89	3.25	0.001	**
Proportion kin in the network	-0.18	0.17	-1.03	0.304	
Social network size	-0.06	0.05	-1.18	0.237	
Mean network education	-0.02	0.10	-0.15	0.879	
Community level variables					
Church in the village (ref = no)	-0.02	0.18	-0.11	0.915	
Health center in the village (ref = no)	-0.49	0.18	-2.74	0.006	**
Mean religiosity	0.00	0.08	0.00	0.998	
Mean education	0.34	0.10	3.32	0.001	***
Variance component					
Between-community variance	0.011				
Model summary and fit					
Sample size	1773				
logLikelihod	-801.40				
DIC	1602.83				

$p < 0.05$*, $p < 0.005$**, $p < 0.001$***

(ii) Artificial methods

Parameter	Estimate	Std. Error	z value	Pr(>|z|)	
Intercept	-6.73	1.58	-4.26	0.000	***
Life history variables					
Parity 0 (ref = no)	-0.72	0.42	-1.72	0.086	.
Parity 1 to 2 (ref = no)	-0.17	0.30	-0.57	0.568	
Parity 3 to 4 (ref = no)	-0.04	0.28	-0.13	0.893	
Parity 5 and above (reference category)					
Age	0.52	0.12	4.42	0.000	***
Age^2	-0.01	0.00	-4.68	0.000	***
Age^3	0.00	0.00	4.33	0.000	***
Marital status (ref = unmarried)	0.16	0.31	0.51	0.613	
Experience of under-5 mortality (ref = no)	-0.22	0.53	-0.42	0.678	
Socioeconomic variables					
Farmer (ref = no)	-0.06	0.17	-0.38	0.702	
Relative education	0.16	0.11	1.53	0.127	
Household material resources	0.32	0.11	2.87	0.004	**
Household market integration	0.09	0.16	0.58	0.562	
Household farming wealth	-0.12	0.09	-1.34	0.179	
Individual sociocultural predictors					
Exposure to social media	0.06	0.12	0.50	0.621	
Ever migrated (ref = no)	0.20	0.14	1.44	0.151	
Relative religiosity	-0.47	0.08	-6.01	0.000	***
Social network and kin variables					
Mother used artificial method (ref =no)	0.24	0.56	0.43	0.668	
Any sister used artificial method (ref = no)	0.52	0.30	1.73	0.084	.
Other kin used artificial method(ref = no)	0.54	0.16	3.35	0.001	***
Number social network alters used artificial method (ref = none)					
1 alter used artificial method	0.65	0.19	3.32	0.001	***
2 alters used artificial method	1.13	0.26	4.41	0.000	***
3 alters used artificial method	1.10	0.39	2.80	0.005	**
4 alters used artificial method	1.58	0.51	3.07	0.002	**
5 alters used artificial method	1.27	0.58	2.18	0.029	*
Proportion kin in the network	-0.02	0.19	-0.11	0.911	
Social network size	-0.02	0.05	-0.38	0.701	
Mean network education	-0.10	0.12	-0.83	0.404	
Community level variables					
Church in the village (ref = no)	0.11	0.19	0.59	0.556	
Health center in the village (ref = no)	0.05	0.18	0.26	0.796	
Mean religiosity	-0.24	0.09	-2.69	0.007	**
Mean education	0.07	0.11	0.68	0.500	
Variance component					
Between-community variance	0.012				
Model summary and fit					
Sample size	1773				
logLikelihod	-732.30				
DIC	1464.70				

$p < 0.05$*, $p < 0.005$**, $p < 0.001$***

Table S8 Global multilevel logistic regression models predicting the odds of (i) any method and (ii) artificial methods of contraception, including mean network education as a control.

In the social network and kin models, we controlled for structure and composition of the social network (i.e. size and proportion of kin), because these are assumed to be sources of bias in the estimation of social network effects on contraceptive behaviour [10], but we did not control for network density, as this results in the exclusion of women with only one or no network partners, reducing the sample by over one quarter to 1,516. Since this means comparisons between women with and without network partners cannot be made it biases the sample towards women with at least two network partners. We ran additional analyses to check whether our social network effects would hold among the subset of women for whom we could calculate network density (n = 1,516), but this predictor was neither significant nor affected the model outcomes (Table S7)

We also examined whether our network results were driven by homophily along educational lines, which would indicate that clustered contraceptive behaviour is driven by educational similarities among network partners, rather than being the result of social transmission among network partners. However, including average network education (which reduced the sample to 1,773) as an additional control had no effect on the model outcomes (Table S8).

We re-ran our model comparisons using a different information criterion, AICc, to see if this affects the model comparison outcomes (Tables S9 and S10). We also examined model comparisons where we first 'dredged' the variables within model classes, i.e. reduced each model to its most parsimonious form before comparison (Tables S11 and S12). All criteria point to the same models being the best approximating models given the data.

As with all cross-sectional analysis, we cannot establish the direction of causality in the models presented here and it is possible that other unobserved factors are important in driving contraceptive uptake. Our models show that contraceptive use is strongly clustered in families and social networks, however we cannot rule out the possibility that individuals simultaneously adopted contraception without making use of any social information from network partners. Longitudinal data are the gold standard for testing hypotheses about the timing of contraceptive uptake and inferring the order in which individuals may influence each other, and since many of the variables used in this analysis could be transformed into a person-period format suitable for such an analysis, a future goal is to replicate this study using a time-to-event approach.

(i) Any Methods

Model	k	logLik	AICc	ΔAICc	ω_i
LH+SN	20	-955.53	1951.5	0.00	1.00
LH+SES	15	-1006.54	2043.3	91.85	0.00
LH+IS	13	-1012.09	2050.4	98.89	0.00
LH+CL	14	-1029.19	2086.6	135.11	0.00
LH	10	-1042.06	2104.2	152.74	0.00

(ii) Artificial methods

Model	k	logLik	AICc	ΔAICc	ω_i
LH+SN	20	-843.07	1726.6	0.00	1.00
LH+IS	13	-875.41	1777.0	50.45	0.00
LH+SES	15	-887.80	1805.8	79.28	0.00
LH+CL	14	-905.58	1839.4	112.81	0.00
LH	10	-916.96	1854.0	127.48	0.00

Table S9 Model comparison and ranking estimates using AIC instead of DIC.

(i) Any Methods

Model	k	logLik	AICc	ΔAICc	ω_i
LH+SES+IS+SN+CL	32	-915.59	1896.3	0.00	0.99
LH+SES+IS+SN	28	-924.64	1906.1	9.85	0.01
LH+IS+SN+CL	27	-926.28	1907.3	11.07	0.00
LH+SES+SN+CL	29	-925.51	1909.9	13.64	0.00
LH+IS+SN	23	-935.63	1917.8	21.56	0.00

(ii) Artificial methods

Model	k	logLik	AICc	ΔAICc	ω_i
LH+SES+IS+SN+CL	32	-795.57	1656.2	0.00	0.90
LH+IS+SN+CL	27	-803.36	1661.5	5.27	0.07
LH+SES+IS+SN	28	-803.02	1662.9	6.66	0.03
LH+IS+SN	23	-811.79	1670.2	13.94	0.00
LH+SES+SN+CL	29	-818.78	1696.5	40.24	0.00

Table S10 Model comparison and ranking estimates based on all possible combinations of model classes (32 possibilities) using AIC instead of DIC.

(i) Any Methods

Model	k	logLik	AICc	ΔAICc	ω_i
LH+SN	19	-956.95	1952.3	0.00	1.00
LH+SES	14	-1008.65	2045.5	93.23	0.00
LH+IS	13	-1012.09	2050.4	98.08	0.00
LH+CL	12	-1029.24	2082.6	130.35	0.00
LH+1	10	-1042.06	2104.2	151.93	0.00

(i) Artificial Methods

Model	k	logLik	AICc	ΔAICc	ω_i
LH+SN	20	-843.07	1726.6	0.00	1.00
LH+IS	13	-875.41	1777.0	50.45	0.00
LH+SES	14	-887.95	1804.1	77.55	0.00
LH+CL	12	-905.89	1835.9	109.37	0.00
LH+2	10	-916.96	1854.0	127.48	0.00

Table S11 Model comparison and ranking estimates based on model classes that have undergone a first round of within-class model selection, i.e. when each model has been reduced to the most parsimonious set of variables.

(i) Any Methods

Model	k	logLik	AICc	ΔAICc	ω_i
LH+SES+IS+SN+CL	23	-922.73	1892.0	0.00	0.99
LH+IS+SN+CL	20	-931.35	1903.1	11.10	0.00
LH+SES+SN+CL	21	-930.47	1903.4	11.40	0.00
LH+SES+IS+SN	21	-931.84	1906.1	14.13	0.00
LH+IS+SN	18	-940.27	1916.9	24.88	0.00

(ii) Artificial methods

Model	k	logLik	AICc	ΔAICc	ω_i
LH+SES+IS+SN+CL	24	-799.52	1647.7	0.00	0.89
LH+IS+SN+CL	20	-805.75	1651.9	4.26	0.11
LH+SES+IS+SN	22	-806.15	1656.8	9.15	0.01
LH+IS+SN	18	-813.15	1662.7	14.99	0.00
LH+SES+SN+CL	21	-823.53	1689.5	41.87	0.00

Table S12 Model comparison and ranking estimates based on all possible combinations of model classes (32 possibilities) that have undergone a first round of within-class model selection, i.e. when each model has been reduced to the most parsimonious set of variables.

References

1. Colleran, H., Jasienska, G., Nenko, I., Galbarczyk, A. & Mace, R. 2015 Fertility decline and the changing dynamics of wealth, status and inequality. *Proc. R. Soc. B Biol. Sci.*

2. R Core Team 2014 R: A language and environment for statistical computing. R Foundation for Statistical Computing, Vienna, Austria. URL http://www.R-project.org/.

3. Gelman, A., Su, Y.-S., Yajima, M., Hill, J., Pittau, M. G., Kerman, J., Zheng, T. & Dorie, V. 2013 arm: Data Analysis Using Regression and Multilevel/Hierarchical Models.

4. Bates, D., Maechler, M. & Bolker, B. 2012 lme4: Linear mixed-effects models using S4 classes.

5. Dorie, V. 2013 blme: Bayesian Linear Mixed-Effects Models.

6. R. H. Baayen 2011 languageR: Data sets and functions with 'Analyzing Linguistic Data: A practical introduction to statistics'.

7. Bartoń, K. 2014 MuMIn: Model selection and model averaging based on information criteria (AICc and alike).

8. Chung, Y., Rabe-hesketh, S., Gelman, A., Dorie, V. & Liu, J. 2011 Avoiding Boundary Estimates in Linear Mixed Models Through Weakly Informative Priors. *Berkeley Prepr.* , 1–30.

9. Chung, Y., Rabe-hesketh, S., Gelman, A., Liu, J. & Dorie, V. 2012 A non-degenerate estimator for hierarchical variance parameters via penalized likelihood estimation. *Psychometrica* , 1–34.

10. Kohler, H. P., Behrman, J. R. & Watkins, S. C. 2001 The density of social networks and fertility decisions: evidence from South Nyanza district, Kenya. *Demography* **38**, 43–58.
